# Assessing data availability and research reproducibility in hydrology and water resources

**DOI:** 10.1038/sdata.2019.30

**Published:** 2019-02-26

**Authors:** James H. Stagge, David E. Rosenberg, Adel M. Abdallah, Hadia Akbar, Nour A. Attallah, Ryan James

**Affiliations:** 1 Utah State University, Department of Civil and Environmental Engineering and Utah Water Research Laboratory, Logan, UT 84321, USA; 2 The Ohio State University, Department of Civil, Environmental and Geodetic Engineering, Columbus, OH 43210, USA; 3 The Western States Water Council, Salt Lake City, UT 84107, USA

**Keywords:** Hydrology, Publishing, Research data

## Abstract

There is broad interest to improve the reproducibility of published research. We developed a survey tool to assess the availability of digital research artifacts published alongside peer-reviewed journal articles (e.g. data, models, code, directions for use) and reproducibility of article results. We used the tool to assess 360 of the 1,989 articles published by six hydrology and water resources journals in 2017. Like studies from other fields, we reproduced results for only a small fraction of articles (1.6% of tested articles) using their available artifacts. We estimated, with 95% confidence, that results might be reproduced for only 0.6% to 6.8% of all 1,989 articles. Unlike prior studies, the survey tool identified key bottlenecks to making work more reproducible. Bottlenecks include: only some digital artifacts available (44% of articles), no directions (89%), or all artifacts available but results not reproducible (5%). The tool (or extensions) can help authors, journals, funders, and institutions to self-assess manuscripts, provide feedback to improve reproducibility, and recognize and reward reproducible articles as examples for others.

## Introduction

The science community is broadly interested to improve the reproducibility of research^1–6^. While exact definitions of reproducibility vary^7–14^, there are recent attempts to harmonize definitions^15,16^. One overarching theme of definitions is that reproducibility requires multiple, progressive components such as (i) all data, models, code, directions, and other digital artifacts used in the research are available for others to reuse (hereafter, “availability”^17–19^), (ii) the artifacts can be used to exactly reproduce published results (reproducibility, sometimes called bit or computational reproducibility;^20,21^), and (iii) existing and new datasets can be processed using the artifacts to reproduce published conclusions (replicability). This progression follows the framework laid out out in a new report on reproducibility and replication by the National Science Foundation and the U.S. Department of Education^16^. In this framework, replicability is a higher standard than reproducibility.

Reproducible and replicable scientific work is currently uncommon because of misaligned incentives and poor coordination among authors, journals, institutions, and funding agencies that conduct, publish, and support scientific research^9,22,23^. For example, making artifacts available requires authors to document additional materials^24,25^ and learn new skills and technologies^26^. Authors may worry that shared materials will never be used^10,27^ or that other scientists will scoop them on follow-up studies^28^. Further, universities typically reward peer-reviewed journal publications, rather than data repositories or documentation, while current scientific culture rewards novelty rather than reproducing prior efforts^2,20^.

Several efforts are underway to encourage more reproducible science^9^. Authors can share research materials in a growing number of online repositories such as Github, Figshare, Harvard Dataverse, Dryad, or HydroShare. Institutional libraries are transitioning to offer online repositories to house digital research artifacts^29–32^. Within our fields of hydrology and water resources, recent tools provide environments to store data publicly and allow software to operate on the data as well as create virtual environments that package code, data, and a working operating system to reduce problems of incompatibility^33,34^. Authors can assign digital object identifiers (DOIs) to research packages to create persistent links and use umbrella research licenses to describe the manner in which these digital artifacts and their associated paper may be legally used by others^22^. Additionally, authors can specify the level of reproducibility that readers and reviewers can expect from each publication, for example that a typical reader could easily reproduce the paper’s results. And yet, despite these powerful tools, few authors are making their work available for others to reproduce or replicate.

**Figure 1:**
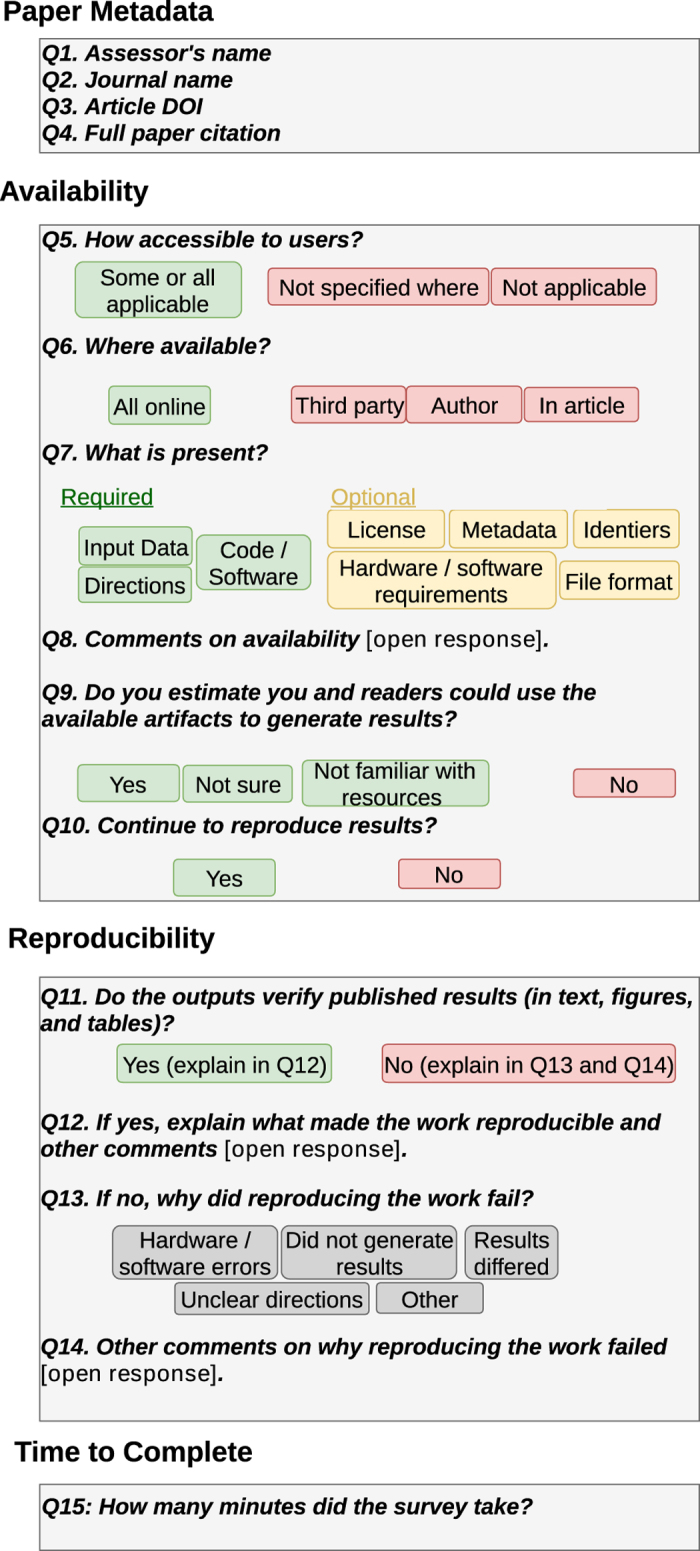
Survey questions to assess journal article data availability and reproducibility. Green and grey answers continue to the next question, while red answers skip to question 15.

To quantify the current state of reproducible science in hydrology and to understand the factors preventing more reproducible or replicable publications, we present here a 15-question survey tool (Fig. 1) designed to assess the availability of digital artifacts and replicability of results in peer-reviewed journal articles (see Methods). We use this survey tool to assess 360 random-sampled articles from the 1,989 articles published in 2017 across six reputable hydrology and water resources journals. The sampling design was stratified by journal and reproducibility keywords of interest to produce a representative population sample, while increasing the linelihood to include articles with reproducible results. Results identify bottlenecks to making digital artifacts available and replicating output. We also use results to generalize reproducibility for the entire sample of articles, test a hypothesis about use of keywords to identify reproducible articles, compare the effectiveness of different journal data availability policies, and highlight how authors, journals, funders, and institutions can use the enclosed survey tool to encourage, recognize, and reward the publication of more reproducible articles.

## Results

**Figure 2:**
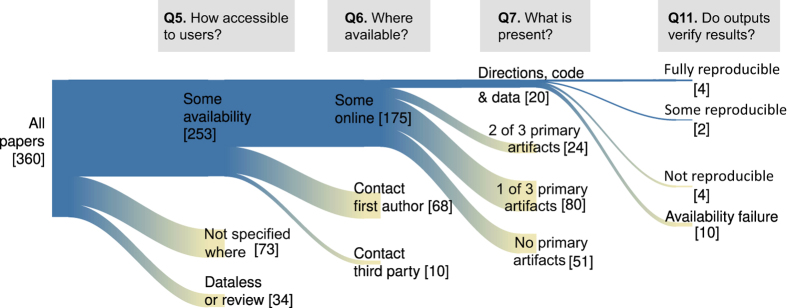
Number of papers progressing through the survey questions to determine availability and reproducibility requirements.

**Figure 3:**
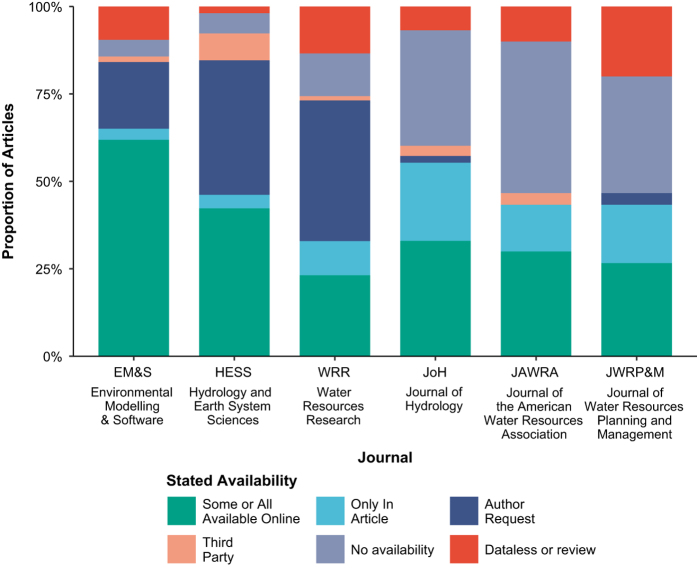
Data, model, code availability by journal (summary of Q4 and Q5).

Applying our survey tool to 360 random-sampled hydrology articles published in 2017 shows that a decreasing number of articles are able to satisfy the progressively stricter reproducibility requirements of artifact availability and ultimately reproduction of the published results (Fig. 2). For example, 70.3% of the 360 sampled articles stated some materials were available, but we could only access 48.6% of those materials online (Fig. 3). Only 5.6% of sampled articles made data, model/code, and directions publicly available while just 1.1% of sampled articles made artifacts available and were fully reproduced. We partially reproduced an additional 0.6% of articles.

### Artifact Availability

**Figure 4:**
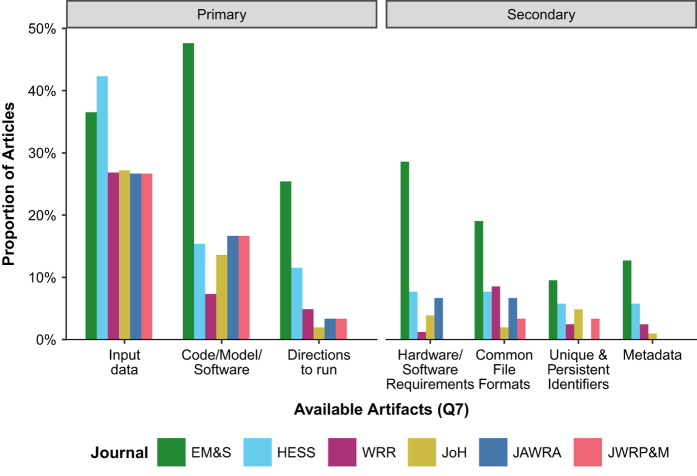
Availability artifacts organized by journal. All percentages are based on the total number of sampled papers for each journal. Refer to Fig. 3 or the text for full journal titles.

Across all sampled publications, the most common primary artifact provided was input data, followed by code/model/software, and then directions to run (Fig. 4). These three primary artifacts were each needed to reproduce modeled results. Secondary artifacts, such as hardware/software requirements, common file formats, unique and persistent digital identifiers, and metadata, were made available at much lower rates than the primary artifacts. Articles published in Environmental Modeling & Software (EM&S) provided data/model/code, directions, hardware/software requirements, common file formats, and metadata at rates two times or higher than other journals.

Sampled articles use different methods to make artifacts available and these methods differ markedly across journals (Fig. 4). A majority of sampled EM&S articles made at least some artifacts available online (61.9%). By contrast, Hydrology and Earth Systems Sciences (HESS) and Water Resources Research (WRR) had high percentages of articles where materials were only available by first author request (38.5–40.2%). Otherwise, the Journal of Hydrology (JoH), Journal of the American Water Resources Association (JAWRA), and Journal of Water Resources Planning and Management (JWRPM) had large proportions of articles where data were available within the article or as supplemental material. These three journals also had high proportions of sampled papers in which research artifacts were not available.

### Reproducibility of Results

Twenty sampled articles (5.6% of total sampled articles) made the required input data, software/model/code, and directions available, allowing an attempt at reproducing published results. We were able to fully reproduce results for four articles^35–38^ and partially reproduced results for two additional articles^39,40^. We were unable to reproduce results for four articles^41–44^, which nonetheless appeared to provide the necessary materials. During the process to reproduce results, we found 10 of the 20 articles did not have all the required artifacts, despite being initially considered for reproducibility testing. Reasons we only partially reproduced results for two articles and did not reproduce results for four articles included unclear directions (4 articles), did not generate results (3 articles), hardware/software error (2 articles), or results differed from the publication (1 article; some articles had multiple reasons). The survey permitted multiple selections for this question. A common issue across cases where we did not generate results was that folder and file locations were hard-coded to work on the author’s computer. If these issues were obvious, we tried, with limited success, to fix them. Other cases pointed to general data gateways, like the USGS streamgauge network, with no further details, or required expensive or proprietary software. Of the 10 articles that had all artifacts available, five were published in EM&S, two articles were published in HESS and in WRR, and the remaining article was published in JWRP&M.

### Estimated Reproducibility for Population

**Figure 5:**
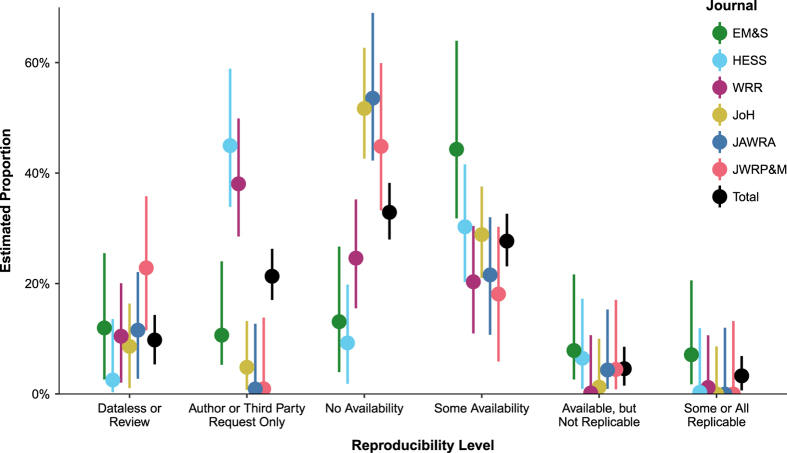
Population estimate of reproducibility for all papers published in 2017. Results are sorted by journal, with “Total” representing all 6 journals. Median estimate is represented by a point, vertical bars show the 95% confidence interval. Refer to Fig. 3 or the text for full journal titles.

Because the stratified sampling method oversampled articles with certain reproducibility keywords, we used bootstrap resampling (see Methods) to estimate that 0.6 to 6.8% of all 1,989 articles published in 2017 in the six journals tested here would be reproducible (95% confidence interval). We estimated 28.0% (23.1–32.6% confidence interval) of all articles published in these journals during 2017 provided at least some of the artifacts necessary for reproducibility (Fig. 5, black). EM&S differed from other journals by having a large proportion of articles with some or all data available (31.8–64.0%) and relatively high estimates of reproducibility (Fig. 5).

### Using Keywords to Identify Reproducible Articles

We found that five of the six articles with some or complete reproducibility had certain related keywords of interest in their abstracts (full list in Methods). This positive hit rate (4.2%) for articles with reproducibility keywords is significantly greater than articles without (0.4%; 2-sample Chi-Squared test with Yate’s continuity correction (p = 0.014)). These findings confirm the value of reproducibility keywords to identify reproducible articles and reaffirm the difficulty to know at the outset whether the results presented in an article are reproducible.

### Time Required to Determine Availability and Reproducibility

**Figure 6:**
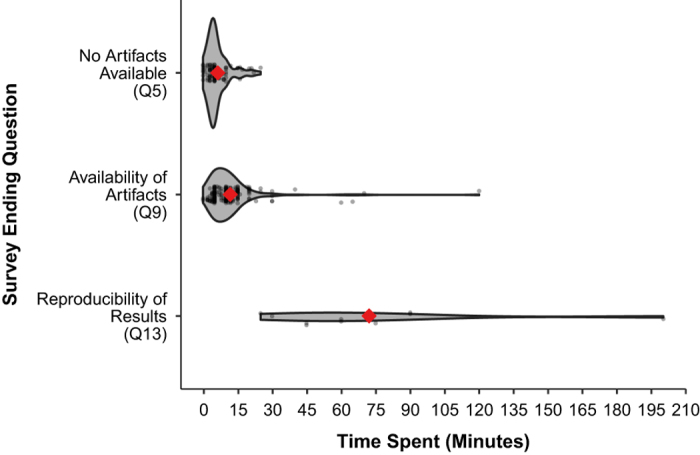
Self-reported time to complete survey organized by the survey’s ending question. Each reviewed paper is shown by a dot, while the mean is represented by a red diamond. Distribution density is shown by width.

We surveyed and analyzed the time required to complete the survey to show the incremental effort required to determine the availability of article artifacts and reproducibility of results (Fig. 6). For example, for a single publication it took us as little as 5 to 14 min (25–75% range) to determine the availability of input data, model/software/code, and directions. Reproducing results for a single paper required upwards of 25 to 86 min (25–75% range), with an upper outlier of 200 min. There were no statistically detectable differences in the time between journals to determine availability of digital artifacts or to reproduce results.

### Reproducibility and Journal Policies

Among the six hydrology and water resources journals we studied, the HESS and WRR policies effective during the 2017 review period require articles to state how data, models, and code can be accessed. In contrast, the 2017 policies by EM&S, WRPM, JoH, and JAWRA simply encouraged this practice. EM&S further recommends articles include an explicit “Software and/or data availability” section within the article and requires authors to make software essential to the paper available to reviewers during the review process (Supplemental Material). HESS includes an assets tab in each publication, based on the Code and Data Availability sections. EM&S, WRR, and JOH are all signatories of the Transparency and Openness Promotion (TOP) policy framwork^45^, while HESS participates in the FAIR (Findable, Accessible, Interoperable, and Reusable) data project^46^.

Stronger journal data availability policies and making open data commitments tend to produce higher rates of artifact availability and result reproducibility. However, there is significant variation among these journals, likely due to minor differences in implementation or other factors. For example, EM&S, which only encourages authors to make artifacts available, had the highest rate of articles that made artifacts available (Fig. 3) and this high rate persisted across nearly every artifact category (Fig. 4). Although EM&S used “should” instead of “must” statements, their policy was by far the most specific for papers with a software component (Supplemental Material). This may explain their high participation rate. EM&S is also a software-focused journal, which may attract papers and authors that are more conscious of reproducible software. In contrast, HESS and WRR, which require data availability statements, had lower percentages of articles that made artifacts available and more papers that direct readers to the authors or third parties for data, models, or code (Fig. 3). These directional statements tend to appear in the Data Availability section of HESS articles and the Acknowledgements of WRR articles. The final group, JoH, JAWRA, and JWRP&M, that also encouraged authors to make artifacts available, had high proportions of articles without available digital artifacts (Fig. 3). The HESS and WRR policies that require data availability statements appear to encourage authors to select options like contact the author rather than work to provide a research article and supporting materials that are available, reproducible, and replicable. In July 2018, JWRP&M switched to start requiring authors to state the availability of data, models, and code, similar to HESS and WRR^47^.

## Discussion

Our findings of low reproducibility of research published in six hydrology and water resources journals in 2017 mirrors low rates of reproducibility previously reported in psychology (100 experiments^2^), computer systems research (613 articles^48^), and articles published in Science (204 articles^6^). Unlike those studies, our survey tool additionally identified bottlenecks to making all digital artifacts available and reproducing results. Here, we discuss how results for our study in hydrology and water resources can inform broader use of the survey tool by authors, journals, funders, and institutions to improve the reproducibility of published scientific research.

### Authors

Authors can use the survey tool as a checklist to self-assess the availability of data, models, code, and directions and reproducibility of their work before submitting work for publication. The tool can help identify missing components that, if provided, will improve reproducibility. For example, our results showed that, for all journals, the number of sampled articles with code/data/software was consistently 2 to 3 times higher than the number of articles that provided directions (Fig. 4). If authors used the tool and subsequently provided directions to use their materials, the tool could potentially double the number of articles which could reasonably be tested for reproducibility. Another bottleneck was a large fraction of authors who chose not to make artifacts available or only made artifacts available by author or third party request. Authors can look to the 10 articles we found that made all digital artifacts available to see easy-to-access platforms to provide access. These platforms included GitHub (6 articles), HydroShare (1 article), journal material (1 article), a custom website (1 article), or Figshare (1 article). Authors who bundled their code and data together in a single GitHub repository further allowed us to download the entire project, with a higher likelihood that code pointed to valid file directories.

### Journals

Journals can use the survey tool to assess the availability of data/model/code and directions, reproducibility of results of new submissions, and provide feedback to authors. Alternatively, journals can require that authors use the survey tool to self-check their work prior to submission. Feedback can be crucial as our study showed that a very low fraction of articles provided all the required artifacts. However, when artifacts were available, we fully or partially reproduced results for 6 out of 10 articles. We also found that time to assess the availability of artifacts was typically less than 15 min, while time to reproduce results was much longer. The combination of these findings suggests that promoting inclusion of digital artifacts through a relatively quick availability survey could pay significant dividends for reproducibility. We leave open whether responsibility for assessment should fall on a journal editor’s assistant, associate editor for reproducibility, reviewers, or others. With a tool to measure reproducibility of published articles, journals could also track reproducibility over time. Tracking and publishing this information would benefit the journal as a promotional tool to show journal commitment to reproducible science. Tracking would also allow journals to acknowledge articles and authors that reach certain reproducibility standards, as implemented by Psychological Science^49^. For example, journals could show a bronze, silver, or gold medal icon on article webpages to recognize and reward progressively greater reproducibility corresponding to availability, reproducibility, and replicability. These badges would simultaneously communicate the expected level of reproducibility of published work. In our study, we are excited to award silver badges to the four articles whose results we fully reproduced^35–38^ and bronze badges to six articles that made all artifacts available^39–44^. This recognition also makes it easier for authors to find and emulate excellent reproducibility practices. We propose these recognition programs as voluntary to encourage authors to make their artifacts available and results reproducible, but not required in cases of proprietary data or code. Cross-journal indices could further aggregate reproducibility metrics and encourage journals and authors to improve the reproducibility of their research portfolios. To oversee these journal efforts, we envision a new role for an Associate Editor of Reproducibility to develop journal data availability and reproducibility policy, manage reproducibility evaluations, and advocate for best reproducibility practices.

### Funders and Institutions

Similar to journals, funders and institutions can use the survey tool to assess artifact availability, verify reproducibility of results, and recognize or reward authors whose work achieves bronze, silver, and gold levels of reproducibility. Alternatively, funders and institutions could use reproducibility assessments made by journals. Funders can encourage authors to use the survey tool to self-check work prior to submitting progress or final reports or use the tool to check the reproducibility of work authors submit. Use of the tool could help verify that author submissions fulfill requirements of funder data management policies and help direct authors to improve the reproducibility of their work. Institutions could also use the survey tool to determine and post the expected level of reproducibility of author work deposited into institutional repositories.

Together, these actions by authors, journals, funders, and institutions can help nudge authors further along the reproducibility continuum to make their digital artifacts more available and to reproduce published results. While these proposed policy nudges represent small shifts targeted at particular actors in the science community, this approach can produce large effects collectively^50^, particularly when all parties agree that the shift will provide a net benefit, as for more reproducible science. Each individual nudge is made possible by using a survey (or similar) tool to measure and quantify the availability of digital artifacts, reproducibility of published results, and replicability of findings. We welcome discussion to improve the survey tool and to improve the reproducibility of our science.

## Methods

### Online Survey Tool

The authors translated definitions of availability, reproducibility, and replicability into a 15-question Qualtrics Research Core (Qualtrics) online survey (Fig. 1). The Qualtrics survey format has been converted into a publicly available Google form, provided here as an example (https://goo.gl/forms/95S4y9BdPmVqMtm02). The survey progressed from soliciting metadata about the article (Questions 1–4), to testing availability of artifacts (Q5–9), and ultimately testing reproducibility of results (Q10–14). Green or yellow shaded answers (Fig. 1) were required to progress to the next question so that survey questions followed the availability and reproducibility progression. Selecting a red-shaded answer stopped progression and directed the reviewer to a final question that asked how many minutes the reviewer spent to reach their stopping point (Q15). This time to complete was self-reported by reviewers rather than using the built-in Qualtrics timer so reviewers could consider the entire time spent reading and assessing the published article and artifacts, rather than the time completing the survey.

The authors developed the tool over four months in Fall 2017 and pre-tested it in early 2018 on a sub-sample of five articles that spanned the availability and reproducibility progression. From our experience pre-testing and to improve use of the tool, we reworded some questions, altered the survey logic, discussed and addressed inter-reviewer variability. Later, after we had reviewed 23% of sampled articles, we added a final question (Q15) to ask how much time it took to complete the survey. We did not re-analyze the time spent for the initial 23% of papers, as reviewers were already familiar with those papers. Instead, we calculated time spent using papers from the remaining sample.

### Selection of Articles

360 peer-reviewed articles were random stratified sampled from the 1,989 articles found in Scopus that were published in 2017 by six well-regarded hydrology and water resources journals. Journals were selected based on impact and to cover a range of hydrology and water resources topics. The six journals were Hydrology and Earth Systems Sciences (HESS), Environmental Modeling & Software (EM&S), Journal of the American Water Resources Association (JAWRA), Journal of Hydrology (JoH), Journal of Water Resources Planning and Management (JWRPM), and Water Resources Research (WRR). Stratified random sampling was approximately proportional to the number of articles published in each journal in 2017, with extra weight placed on articles with a set of reproducibility-related keywords (Table 1).

We further adjusted the stratification so each journal had at least 30 articles (JAWRA and JWRPM were oversampled). Similarly, we oversampled articles with the keywords: analytical software, application programs, C++, cloud computing, computational reproducibility, computer modeling, computer programming, computer software, computer software usability, computer-based models, development and testing, engineering software, fortran, freely available data, freely available software, github, hardware and software, java, open code, open source, replicative validation, scientific software, code, python, cran, and http. Of the 120 articles published in the six journals in 2017 that had at least one keyword, we sampled 119 articles, principally to retain at least 15 non-keyword articles for each journal with an approximately 2:1 non-keyword to keyword ratio overall.

Each author was randomly assigned 60 articles stratified by journal to assess the availability of article artifacts (Q1–9). After identifying all publications that had the available artifacts, we re-assigned reviewers to assess whether the published results could be reproduced (Q1–15). We carried through responses of “Not sure” or “Not familiar with resources” to Q9 and reassigned these articles to match article software with a reviewer most familiar with those tools. The Qualtrics online format allowed us to both simultaneously and asynchronously assess journal articles and store survey responses in an accompanying Qualtrics database. After all availability and reproducibility assessments were complete, we exported results from the Qualtrics database to a text file which was processed in R to generate figures, tables, and results presented in this article. Time spent to complete the survey (Q15) was analyzed for three key stopping points: no artifacts available (Q5), availability of artifacts (Q9), and reproducibility of results (Q13).

### Population Estimates

Resampling was used to estimate the overall percentage of articles from all n = 1,989 articles published in 2017 in the six journals while adjusting for keyword and journal sampling. Sampled articles were sorted into six mutually exclusive categories that were stopping points in the survey: Data-less or review, Author or Third Party Only, No availability, Some availability, Available but not reproducible, and Some or all reproducible. “Some availability” included articles with one or two data/model/code, and direction elements of the three required elements (Q7). “Available but not reproducible” articles had all three required elements available on the initial review, but either could not be reproduced or were found to be missing key elements when reviewers attempted to reproduce the results.

The resampling approach generated 5,000 random populations. Each population had 1,989 articles. In each population, we inserted the 360 articles we manually assessed, assuming that we exactly knew the reproducibility of these articles. Estimates for the remaining 1,629 unsampled articles were simulated based on survey results for the sampled articles in their stratified category, i.e. journal and keyword/non-keyword. For each random sampled population, the proportion of unsampled articles in each reproducibility category was randomly simulated using the multinomial uncertainty approach of Sison and Glaz^51,52^. This produced 5,000 sample populations equal in size and distribution (journal and keyword) to the true population of articles published in 2017, while incorporating uncertainty due to unsampled papers.

### Code Availability

The survey tool, Qualtrics results, and all code used for analysis presented in this article are available online through the permanent repository^53^. Please cite this repository for any use of the related data or code. Additionally, results can be reproduced using RStudio deployed in the cloud using MyBinder through the GitHub website.

### Data Availability

All relevant data presented in this article are available online through the permanent repository^53^. Please cite this repository for any use of the related data or code. An open Google Forms version of the survey tool is available for readers to use, modify, and extend (https://goo.gl/forms/95S4y9BdPmVqMtm02). A pdf image of the survey tool is also available in the permanent repository^53^.

## Additional information

**How to cite this article**: Stagge, J. H. *et al*. Assessing data availability and research reproducibility in hydrology and water resources. *Sci. Data*. 6:190030 https://doi.org/10.1038/sdata.2019.30 (2019).

**Publisher’s note**: Springer Nature remains neutral with regard to jurisdictional claims in published maps and institutional affiliations.

## Supplementary Material

Supplementary Information

## Figures and Tables

**Table 1:** Number of articles published in 2017 and number of articles sampled by journal.

	**EM&S**		**HESS**		**WRR**		**JoH**		**JAWRA**		**JWRP&M**	
	*2017*	*Sample*	*2017*	*Sample*	*2017*	*Sample*	*2017*	*Sample*	*2017*	*Sample*	*2017*	*Sample*
Keyword	49	48	9	9	23	23	24	24	7	7	8	8
Non-keyword	181	15	319	43	511	59	645	79	102	23	111	22
Total	230	63	328	52	534	82	669	103	109	30	119	30
