# Author Correction: Assessing data availability and research reproducibility in hydrology and water resources

**DOI:** 10.1038/s41597-019-0039-0

**Published:** 2019-04-24

**Authors:** James H. Stagge, David E. Rosenberg, Adel M. Abdallah, Hadia Akbar, Nour A. Attallah, Ryan James

**Affiliations:** 1 Utah State University, Department of Civil and Environmental Engineering and Utah Water Research Laboratory, Logan, UT 84321 USA; 2 The Ohio State University, Department of Civil, Environmental and Geodetic Engineering, Columbus, OH 43210 USA; 3 The Western States Water Council, Salt Lake City, UT 84107 USA

Correction to: *Scientific Data* 10.1038/sdata.2019.30, published online 26 February 2019

In the original version of this Article the author of reference #38 was incorrectly stated as Quinn, J. *et al*. This has been corrected to Xu, W. *et al*. in both the HTML and PDF versions.

