# Supplementary material for: Assessing data availability and research reproducibility in hydrology and water resources
Source: Sci Data. 2019 Feb 26;6:190030. doi: 10.1038/sdata.2019.30 (PMC6390703; doi:10.1038/sdata.2019.30)
Supplement: Supplementary Information [file sdata201930-s1.pdf]

SCIENTIFIC DATA

Supplementary Material

Table S1: Summary of journal data policy snapshots in the selected water resources journals as of December 2017. Key words indicating the level of the requirement is highlighted in bold.

**Journal**	**Data Policy Statement**	**URL**
Environmental Modeling & Software (EM&S)	Where appropriate, a software/data availability section **should** be provided, containing as much of the following information as possible ...<br><br>When a software component is an essential part of the paper presentation, authors **should** be prepared to make it available to reviewers during the review process. To preserve the anonymity of reviewers, the authors **should** make the software available for a download.	`https://www.elsevier.com/journals/environmental-modelling-and-software/1364-8152/guide-for-authors`
Hydrology and Earth Systems Science (HESS)	Each manuscript **must** have a final Data Availability section that states how the underlying research data can be accessed.	`http://www.hydrology-and-earth-system-sciences.net/about/data_policy.html`
Water Resources Research (WRR)	AGU's Data Policy states that all data necessary to understand, evaluate, replicate, and build upon the reported research **must** be made available and accessible whenever possible.	`http://publications.agu.org/author-resource-center/publication-policies/data-policy/`
Journal of the American Water Resources Association (JAWRA)	JAWRA **encourages** authors to share the data and other artefacts supporting the results in the paper by archiving it in an appropriate public archive.	`https://onlinelibrary.wiley.com/page/journal/17521688/homepage/forauthors.html`
Journal of Hydrology (JoH)	To foster transparency, we **encourage** you to state the availability of your data in your submission.	`https://\www.elsevier.com/journals/environmental-modelling-and-software/1364-8152/guide-for-authors`
Journal of Water Resources Planning and Management (JWRPM)	Authors are **encouraged** to use Supplemental Data to show all necessary data	`https://ascelibrary.org/doi/pdf/10.1061/9780784479018.ch03`
